# eMouseAtlas: An atlas-based resource for understanding mammalian embryogenesis

**DOI:** 10.1016/j.ydbio.2017.01.023

**Published:** 2017-03-01

**Authors:** Chris Armit, Lorna Richardson, Shanmugasundaram Venkataraman, Liz Graham, Nicholas Burton, Bill Hill, Yiya Yang, Richard A Baldock

**Affiliations:** MRC Human Genetics Unit, Institute of Genetics and Molecular Medicine, University of Edinburgh, Crewe Road, EH4 2XU, UK

## Abstract

The eMouseAtlas resource is an online database of 3D digital models of mouse development, an ontology of mouse embryo anatomy and a gene-expression database with about 30K spatially mapped gene-expression patterns. It is closely linked with the MGI/GXD database at the Jackson Laboratory and holds links to almost all available image-based gene-expression data for the mouse embryo. In this resource article we describe the novel web-based tools we have developed for 3D visualisation of embryo anatomy and gene expression. We show how mapping of gene expression data onto spatial models delivers a framework for capturing gene expression that enhances our understanding of development, and we review the exploratory tools utilised by the EMAGE gene expression database as a means of defining co-expression of *in situ* hybridisation, immunohistochemistry, and lacZ-omic expression patterns. We report on recent developments of the *eHistology* atlas and our use of web-services to support embedding of the online ‘The Atlas of Mouse Development’ in the context of other resources such as the DMDD mouse phenotype database. In addition, we discuss new developments including a cellular-resolution placental atlas, third-party atlas models, clonal analysis data and a new interactive *eLearning* resource for developmental processes.

## Highlights

• 3D Atlas models enable detailed understanding of mouse embryo anatomy.
• Point clouds facilitate enhanced visualisation of coexpression.
• Database queries allow gene expression patterns to be analysed *spatially* in relation to each other.
• Web-services enable Atlas support for phenotyping studies.

## 1 Introduction

Open access online atlases and databases are critical in managing the rapidly growing amount of data describing embryogenesis. A recent review (Clarkson, 2016) described multiple criteria that are used by online atlas resources to present stage-focused information, anatomical information, and to provide text and spatial methods for querying gene expression. Importantly, this review stressed the importance of using annotation terms that conform to a community standard ontology, and of supporting these terms with visual definitions. eMouseAtlas utilises these core concepts in providing a comprehensive resource that enables a deep understanding of embryogenesis.

Digital Atlas frameworks play a major role enabling researchers to collate, compare and analyse data and to understand developmental processes. Through visualizing development as a series of discrete developmental stages, it is possible to define developmental trajectories and to detect when and where critical changes occur during embryogenesis. The eMouseAtlas web-resource provides 3D digital atlas models of mouse embryos at a series of morphologically distinct stages of development plus a large-scale database of spatially mapped gene-expression patterns. This resource makes full use of volume-rendered and surface-rendered 3D reconstructions that have been described in a series of articles (Armit et al., 2012, Armit et al., 2015, Christiansen et al., 2006, Richardson et al., 2010, Richardson et al., 2014). Here we extend those publications to report on new 3D tools, new data types and the community support for other mouse embryo atlases such as the OPT limb atlas (deLaurier et al., 2008) and Caltech MRI models (Ruffins and Jacobs, 2011).

A major feature of the EMAGE^1^ gene expression database is the use of atlas models as a spatiotemporal framework for capturing gene expression patterns. We use direct spatial mapping of image data for inclusion in the EMAGE database. This is critical as gene expression patterns do not necessarily correspond to named anatomical structures and can be difficult to describe using only text-based annotation. Recently, we have developed and made freely available the *eHistology* resource (Graham et al., 2015) as a means of capturing cellular-resolution anatomical and histological detail. This resource utilises the sections and annotations originally used in the ‘The Atlas of Mouse Development’ (Kaufman 1994), and makes this data available through a web-accessible pan-and-zoom interface. These high-resolution sections now include new coronal sections from “Kaufman's Atlas of Mouse Development Supplement” (Baldock et al., 2015). The 3D anatomy atlas, the EMAGE database, and the *eHistology* atlas are fully integrated through use of a common EMAPA anatomy ontology framework (Hayamizu et al., 2015), which enables queries to be performed easily across eMouseAtlas resources and other resources such as the GXD gene expression database at the MGI, Jackson Laboratory (Smith et al., 2014, Smith et al., 2015).

To date the 3D models could be displayed online using the IIP3D viewer (Husz et al., 2012), which provides virtual sections through the 3D volume at any viewing orientation. The anatomy was made visible as an interactive overlay on the underlying histological image. We have extended this visualisation to include interactive rendering in a 3D window sharing the same anatomy controls as the section-viewer (see Section 2). This visualisation uses the WebGL Javascript library three.js (threejs.org) and includes the rendering of complex gene-expression patterns as “point-clouds” (see Section 3). In Section 4 we describe new data-types now available from EMAGE including gene and regulatory expression patterns and the results of clonal analysis encoding fate maps and lineage and in section 5 the recent developments linking the *eHistology* resource to the high-resolution phenotype data now analysed and published by the Deciphering Mechanisms of Developmental Disorders (DMDD) on-line resource. In section 6 we describe the new *eLearning* resource providing interactive vignettes of developmental processes based on the on-line tutorial materials from José Garcia Monterde (http://www.uco.es/organiza/departamentos/anatomia-y-anat-patologica/embriologia/MyWeb_i/index.html).

## 2 Web-based 3D visualisation of anatomy

**Fig. 1:**
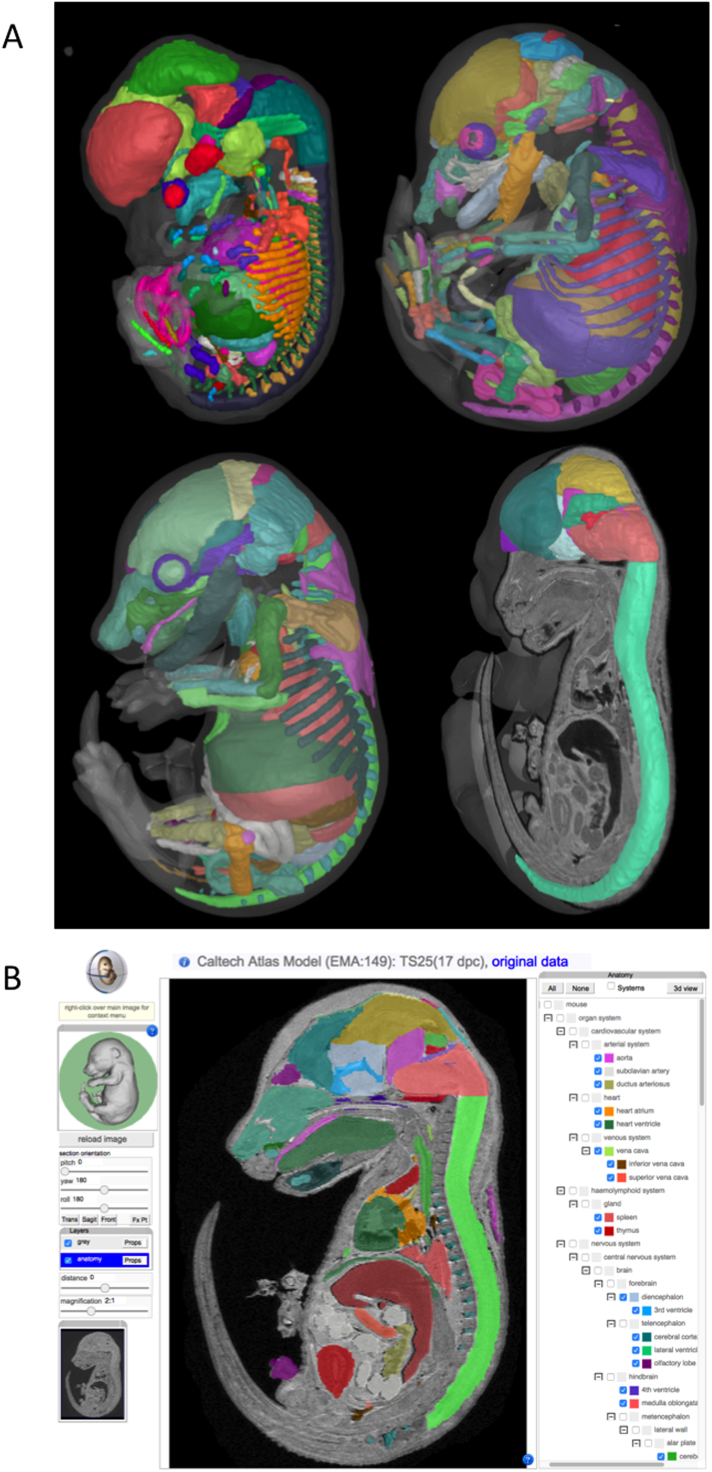
3D atlas models visualised using the eMouseAtlas 3D atlas viewer. A) Surface-rendered views of Theiler stage 21 (top left), Theiler stage 24 (top right), and Theiler stage 25 (lower left and right) mouse embryo models with delineated anatomy can be interactively explored using a web browser. B) The eMouseAtlas IIP3D section viewer allows users to define an arbitrary section through a 3D volume using a web browser. Navigation controls are provided in the left panel and include: pan-and-zoom; translating the viewing plane (distance); and rotating the viewing plane in 3 dimensions (pitch, yaw). The central panel shows a section through a 3D model with delineated anatomy shown in various colours. An anatomy tree in the right panel is used to visualise delineated anatomical domains. Clicking on 3D view (top right) opens up the selected anatomical domains as a surface rendered object in a separate window*.*

Developments in 3D visualisation standards for web-browsers based on WebGL/HTML5 are now implemented and available for all widely used browsers and operating systems. We have utilised this capability to provide an interactive 3D viewer showing a surface rendering of the defined anatomic components (Fig. 1a). This supercedes the Java-based viewer we had implemented previously (Feng et al., 2005), which, with the deprecation of Java embedded applications for the web, was not maintained. The new visualisation is controlled by the anatomy tree provided in the section browser (Fig. 1b) and allows selection of visibility, colour and transparency of the displayed structures. There is the additional option to view in wireframe mode for the larger models, which may display slowly. We have enabled this functionality in all our embryo models and so provide 3D anatomy of the developing mouse from implantation, through gastrulation and neurulation, and into mid- and late-stage embryogenesis.

Uniquely, the section viewer allows visualisation of anatomy on a section of *arbitrary orientation* through mouse embryos. Specific anatomical components can be toggled *on* or *off* through their selection in the anatomy tree or by clicking directly on a component displayed in the section of interest. Anatomy components selected in this way are automatically displayed in the 3D anatomy viewer including any colour selections. An important feature of the section viewer is its ability to deliver sections through very large 3D volumes without the need for data download. This circumvents a common issue with many 3D viewer applications which require download of very large 3D volumes. IIP3D technology allows a single section through a 3D volume to be delivered and displayed, and consequently retrieving a section image is fast and can be accomplished on large volume 3D images (Husz et al., 2012).

The anatomy delineation process was undertaken by developmental biologists and anatomists and has involved delineation of visible anatomical components on a section-by-section basis through the 3D reconstruction of Theiler-staged mouse embryos. To support the delineated anatomy, a mouse anatomy ontology was created that enabled the partonomic (*part-of*) relationships of mouse developmental anatomy to be rationalized and to be queried through computational means. To annotate the anatomy in the 3D reconstruction, each delineated anatomical component was assigned to a specific component in the anatomy ontology, and this enables the tree-view display that can be seen in the section viewer in the context of an interactive anatomy tree that displays the *part-of* relationships as sub-branches of the various anatomical components. This process of delineation allows the 3D anatomy viewer to provide precise context for understanding mouse embryo development, and enables interoperability between the 3D atlas and the *eHistology* and EMAGE database resources such that an annotated component can be visualised in 3D alongside whole-mount and section data. This interoperability allows for visualisation and communication of the relationship between gene expression and anatomy.

## 3 3D visualisation of molecular anatomy

**Fig. 2:**
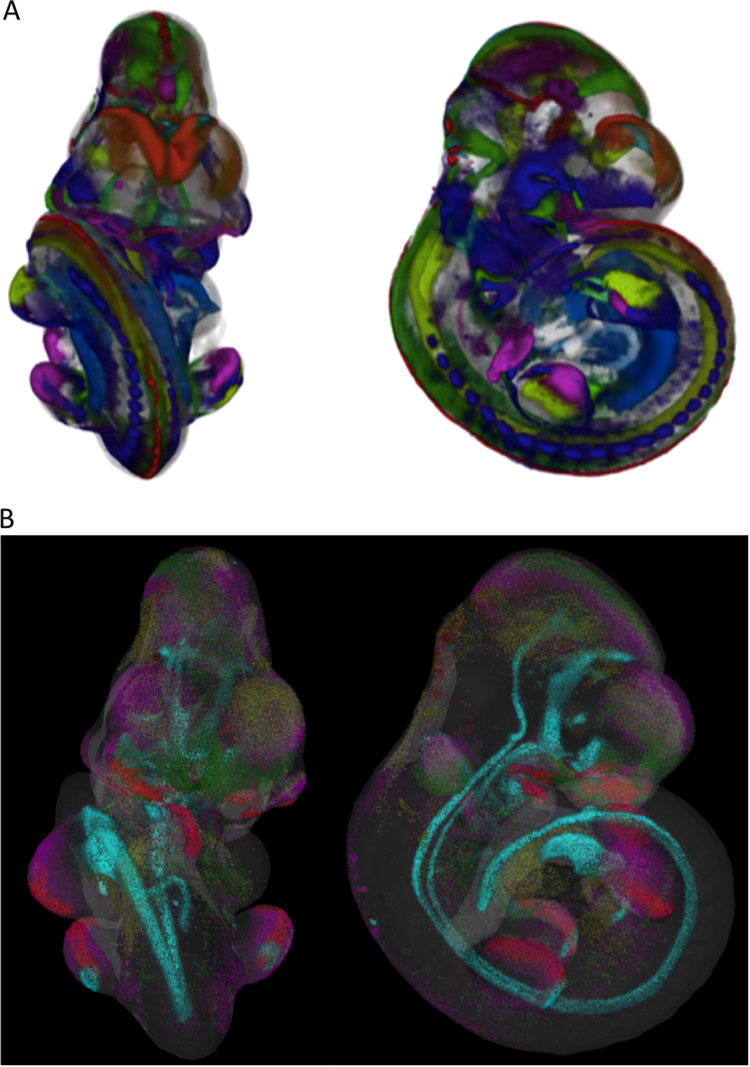
3D visualisation of molecular anatomy. A) Volume rendered views of 19 E10.5 (TS17) spatially mapped Wnt family gene expression patterns can be generated using commercially available software packages such as Amira. These visualisations can be used to cross-compare gene expression patterns in a spatial framework. B) Point-cloud rendered views of spatially mapped E10.5 (TS17) Shh (cyan), Fgf8 (red), Fgf9 (green), Fgf10 (magenta), and Fgf20 (yellow) enable direct visualisation of spatially mapped gene expression patterns in the context of a web browser and without the need for data download. An advantage of the point cloud visualisation is that a user can see through the entire 3D volume, and can more accurately identify regions of expression. An interactive point cloud visualisation can be found at the following link: http://aberlour.hgu.mrc.ac.uk/MARenderTests/genexpr-shh+fgf.html.

A primary objective of the eMouseAtlas Project was to enable spatial mapping of whole embryo gene expression patterns to allow complex 3D gene expression patterns to be visualised, indexed, cross-compared and to deliver objective analysis using spatial analysis tools. The tools developed for internal use (WoolzWarp) use a novel 3D warping technique based on the constrained distance transform (Hill and Baldock, 2015), which allows interactive 3D mapping of the full image volume. The mapping process utilises points of morphological equivalence that are defined on the source gene-expression data image and the stage-matched embryo model. These points define the spatial warp that maps the original data image into the space of the reference model. Having defined the transform, the signal in the original image is extracted and a representation of the original pattern is defined within the space of a 3D atlas model, without the constraint of anatomical boundaries. Mapping gene expression patterns in this way provides an objective and accurate representation of the whole embryo expression patterns and additionally captures information on expression gradients. Multiple mapped expression patterns can then be visualised and cross-compared in a common spatial framework (Fig. 2a).

3D visualisation enables understanding of co-expression in a 3D context, but additionally informs our understanding of gene expression boundaries and the interlocking nature of gene expression patterns in what are in essence molecular profile-defined anatomical regions in the developing mouse embryo. In Fig. 2a we provide an example where 3D mapping enables *Wnt* family gene expression to be visualised in a 3D spatial model. Direct 3D visualisation of the expression profiles of all 19 *Wnt* family genes highlights the complexity of specific compartments of the embryo in terms of *Wnt* coexpression. Tools are needed to isolate coexpression domains in these complex 3D models, and these represent an important future development for this aspect of the project. In addition, there are key advantages in being able to sequentially order gene expression in compartments of the developing mouse embryo in these rich spatial datasets. For example, tools that would enable nested sets of gene expression and other hitherto poorly understood spatial relationships to be identified in these 3D models of gene expression may help us understand the regulation of *Wnt* gene expression.

From a visualisation perspective, mapped gene expression has additional complexities that we do not encounter with anatomical domains. In contrast to the 3D anatomy, where computational measures were used to ensure that overlaps did not occur, the molecular anatomical gene expression profiles in general overlap in 3D voxel-space and our core measure of putative coexpression can be calculated using the Jaccard index (Armit et al., 2015). To be able to identify whether genes are coexpressed (overlap) or show adjacent expression domains (non-overlap) we have developed a viewer that enables point cloud visualisation of the 3D molecular anatomy as this enables both overlapping and non-overlapping gene expression domains to be explored, and overcomes unwanted colour-mixing effects that are a feature of surface-rendered overlapping domains. In Fig. 2b we provide an example of a point cloud visualisation of *Fgf* genes and *Shh*. In this example, it can be seen that a major advantage of using the point cloud visualisation is the ability to see through the entire mapped 3D volume.

From a data mining perspective, we are additionally exploring computational measures that can be used to define adjacency, and to determine whether non-overlapping domains are coupled in other ways, for example whether they track one another longitudinally along the alar and basal plates of the developing CNS. It is anticipated that the 3D modelling and 3D spatial analysis approach outlined here will enable a deeper understanding of the role of genes in development, and may deliver powerful new tools that will allow researchers to explore the cross-regulation of key signal transduction pathways such as *Wnt*, *Shh, Notch,* and *Fgf* signaling pathways in the developing mouse embryo.

## 4 Gene and regulatory element expression patterns in the EMAGE Database

The 3D atlas framework is a resource describing and defining the developmental anatomy of the mouse and was originally developed as a spatio-temporal framework for capturing spatially organised image data, specifically *in situ* gene-expression. This is the basis of the Edinburgh Mouse Atlas Gene Expression (EMAGE) Database and provided a mechanism to index and collate spatial *in situ* patterns in an unbiased form and independent of the constraints of the underlying anatomical structures. The initial data captured was *in situ* gene-expression revealed by hybridisation to mRNA (ISH), and this was soon extended to include immunohistochemistry (IHC) and transgene reporter (ISR) expression patterns. All of these data are directly mapped to the 2D and 3D embryo models to enable spatial indexing, query, cross comparison and analysis. The mapping process involves accurate stage selection of mouse embryo models and *spatial warping* of raw image data onto a stage-matched model (described in Section 3 and reviewed in Armit et al. (2015)).

**Fig. 3:**
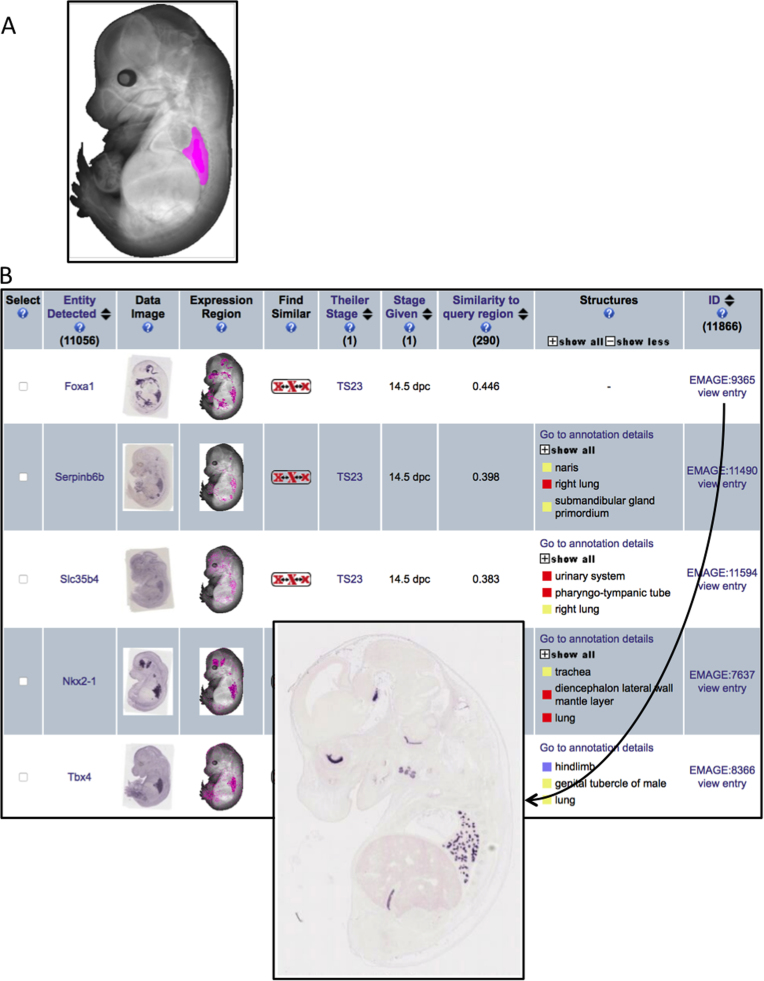
EMAGE Spatial Query. A use case scenario in which an EMAGE spatial query is used to identify gene expression patterns in the developing lung. A) A user-defined region (pink) on an embryo model is delineated using the Embryo Space paint query tool. B) A results table returns a list of EMAGE entries ranked by spatial similarity to the defined region of interest. The gene symbol (entity detected), spatial annotation (expression region), text annotation (structures), and similarity score (similarity to query region) are all shown in the results table. High-resolution original images (inset) and probe details are found on the EMAGE entry page, which can be accessed by clicking on the EMAGE entry ID in the results table. This example query identified *Foxa1*, *Serpin6b*, *Slc35b4*, *Nkx2-1*, and *Tbx4* as the top 5 genes that show spatially mapped expression in the E14.5 (TS23) lung. The first gene in this list, *Foxa1*, was not text-annotated by the authors but was spatially mapped by the EMAGE Editorial Office and so could be retrieved by spatial query.

This mapping process generates standardised representations of gene expression patterns that can be archived in the EMAGE database, and queried through a web interface. New discoveries can be extracted using this web interface, and in Fig. 3 we highlight a use case scenario in which an EMAGE spatial query is used to identify gene expression patterns in the developing lung. This example query identified *Foxa1*, *Serpin6b*, *Slc35b4*, *Nkx2-1*, and *Tbx4* as the top 5 genes that show spatially mapped expression in the lung. Importantly, the first gene in this list – *Foxa1* – was not text-annotated by the authors and so it would be impossible to find this gene through a text-based query for genes expressed in the lung. An additional advantage of this system is that expression patterns can be analysed *spatially* in relation to each other, allowing gene expression patterns to be ranked and clustered through computational measures of spatial similarity. The EMAGE database archives ISH, IHC, and ISR gene expression patterns from a near-comprehensive set of 17,554 genes over 22 stages of post-implantation development. Each EMAGE entry details the expression pattern of a single gene at a single stage of development, and may include multiple images of an embryo specimen. Over 15 years, the EMAGE database has accumulated a total of 32,248 entries and 427,691 images, and includes ISH data generated from important screens including Eurexpress, EmbryoExpress, and EMBRYS. These spatial data-types have been extended to include regulatory element expression patterns from the VISTA and TRACER resources and clonal analysis data encoding lineage relationships.

VISTA is a resource of experimentally validated human and mouse non-coding genomic DNA fragments with gene enhancer activity as assessed in transgenic mice (Visel et al., 2007). Enhancer candidate sequences were identified by “extreme” evolutionary sequence conservation or by ChIP-seq and were labelled with lacZ to reveal the enhancer expression pattern in mouse embryos. The EMAGE editors have spatially mapped the VISTA murine enhancer lacZ-omic dataset (Richardson et al., 2014) enabling comparisons to be made between putative mouse enhancer elements and *in situ* expression patterns. Here we announce the spatial mapping of the TRACER dataset of transposon-associated regulatory sensors (Chen et al., 2013). This resource utilises Sleeping Beauty lacZ transposons that have been randomly integrated into the mouse genome. Hundreds of insertions have been mapped to specific genomic positions, and the corresponding regulatory potential is documented through lacZ imaging of E11.5 wholemount mouse embryos. Through spatial mapping of the lacZ expression patterns, the EMAGE gene expression database enables rapid identification of *cis*-regulatory elements that are expressed in a region of interest in the mouse embryo. Additional query capabilities - such as the *find similar* query - enable co-localisation and co-expression of regulatory elements to be explored computationally and allow the VISTA and TRACER datasets to be spatially compared with ISH gene expression profiles.

Lineage tracing is a powerful means of describing recruitment of cells to various tissue compartments (reviewed in Wilson and Lawson (2015)). Importantly, studies of clonal analysis that utilise labelling of individual cells enrich our understanding of developmental processes and can reveal cryptic boundaries that may exist within the developing embryo. We have collaborated with Drs Val Wilson and Kirstie Lawson of the University of Edinburgh to develop a clonal analysis database that allows researchers to explore patterns of genetic mosaicism that have been uncovered in the mid-gestation mouse embryo. Dr Val Wilson's group have utilised a single-cell labelling method that relies on the spontaneous reversion of an inactive lacZ gene (laacZ) carrying a sequence duplication to an active lacZ reporter (Tzouanacou et al., 2009). Spontaneous reversion is a rare recombination event, and clones derived from laacZ/lacZ-revertant progenitor cells reveal lineage relationships between seemingly disparate embryonic tissues. In addition, the size of the clones allow for estimation of the time at which the progenitor cells were labelled. To generate EMAGE entries for this dataset, we have created a mapping between anatomical terms used by the annotators and the closest match in the EMAPA ontology. This step ensures EMAGE anatomical queries will return these data entries. In addition, a mapping between the annotator's in-house scoring criteria and the EMAGE nomenclature was used to describe whether clones are *present* or *not detected* in a specific anatomical component.

**Fig. 4:**
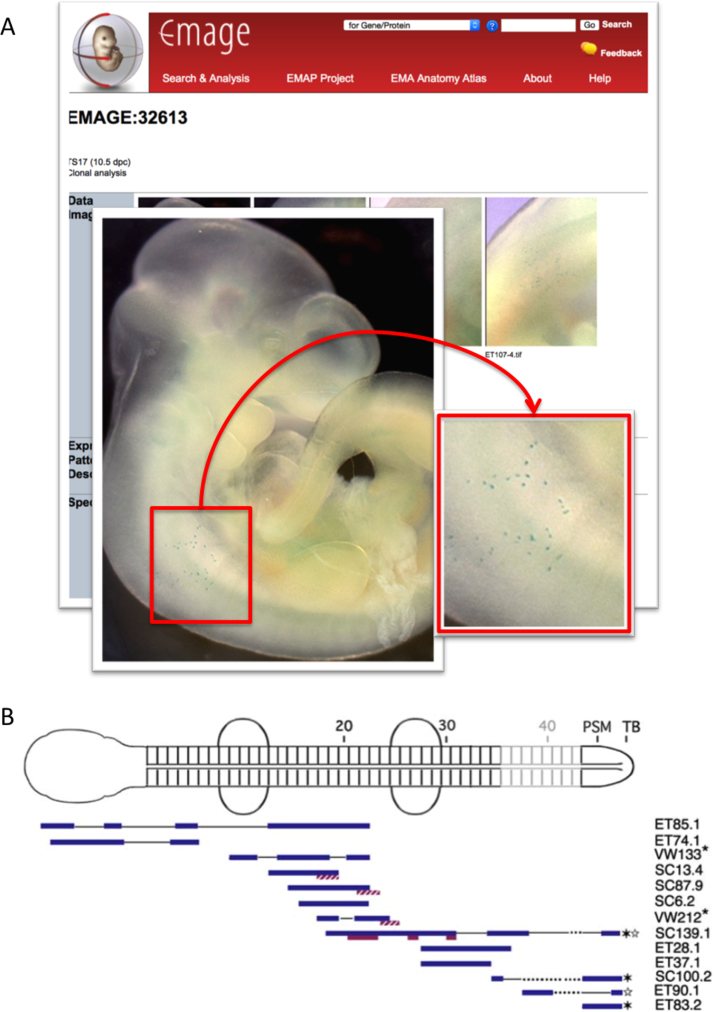
A spatial model for capturing clonal analysis data. A) Clonal analysis data is hosted by the EMAGE database. These entries include text-annotation provided by the original authors. B) Clonal analysis maps used by the authors as part of their annotation process can additionally be incorporated into EMAGE entries and used to capture clone distribution. Reprinted from Fig. S4 in Developmental Cell 17, Tzouanacou et al., Redefining the progression of lineage segregations during mammalian embryogenesis by clonal analysis, 365-76, Copyright (2009), with permission from Elsevier.

In future developments we wish to develop clonal maps with the aim of capturing clone distribution in a spatial model. Dr Val Wilson's group have captured this spatial information as part of their annotation process (Fig. 4) and by archiving these data in a spatial model we will be able to analyse these data *spatially* in relation to each other, allowing clonal distribution to be ranked and clustered through computational measures of spatial similarity.

## 5 *eHistology* and mouse embryo phenotyping

The *eHistology* resource provides online access to cellular-resolution images of the histology sections used in Kaufman's *The Atlas of Mouse Development* (Graham et al., 2015). The original publication of this book by Kaufman (1992) is the definitive work for mouse developmental anatomy. Here we have re-digitised, in high-resolution and colour, the original H&E stained histology sections on glass slides and in collaboration with Elsevier made the new images freely available on-line with a zoom-viewer. These new images show the >10,000 annotations from the original paper atlas and link them to terms used in the mouse anatomy ontology (reviewed in Richardson et al. (2015)). Mapping the annotations in this way enables queries to be performed from *eHistology* across EMAGE and GXD databases.

The *eHistology* resource has now been extended to include the annotated coronal sections published in Kaufman's *Atlas of Mouse Development Supplement* (Price et al., 2015). This dataset catered for a specific demand from the neuroscience community for annotated coronal sections that could be used as a reference atlas, and includes detailed annotation of the brain at E11, E11.5, E12.5, E13.5, E14.5 and E15.5. These sections include some original annotations from M.H. Kaufman supplemented by more detailed brain annotations.

**Fig. 5:**
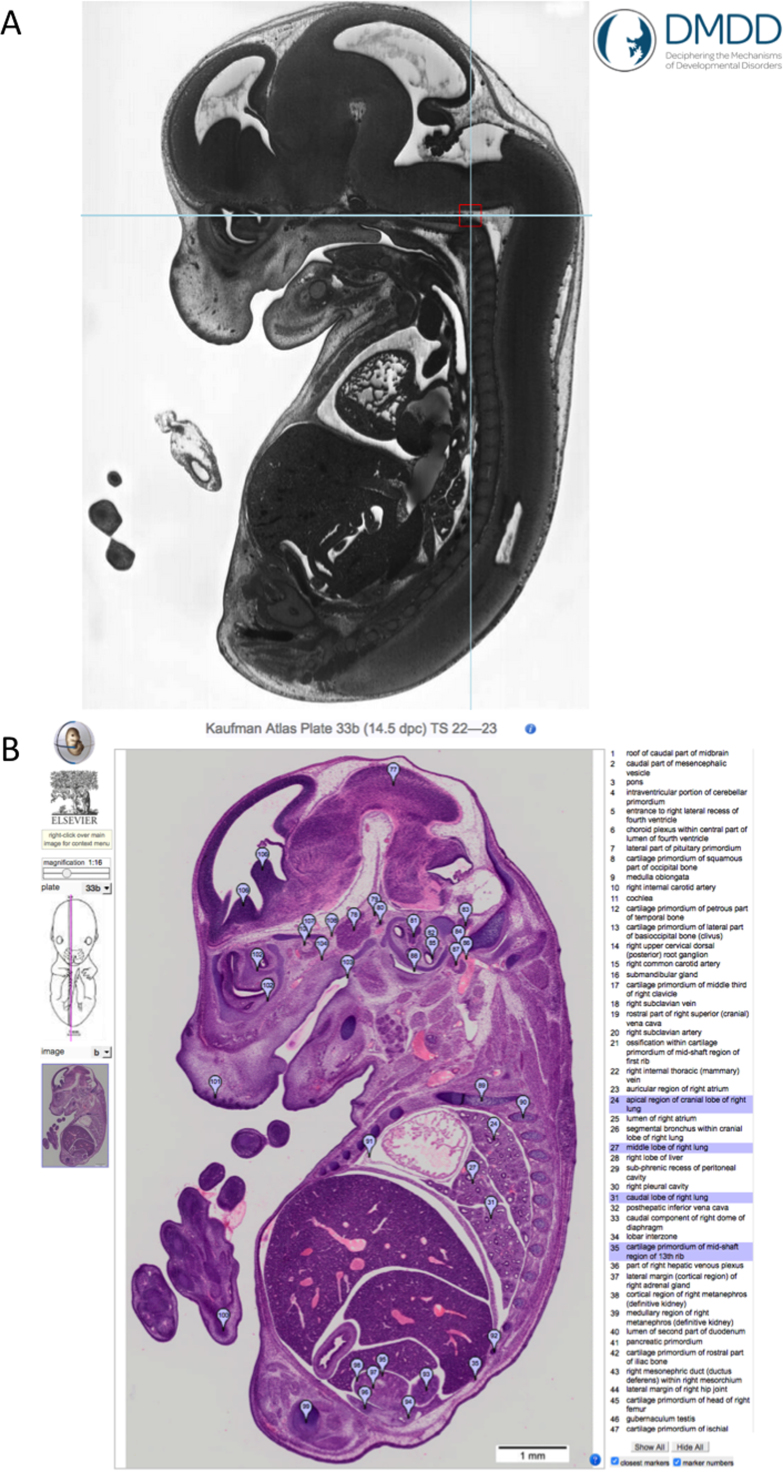
eHistology and phenotyping. eHistology supports the DMDD phenotyping effort by providing detailed annotation to accompany phenotype image data. A) The DMDD web resource hosts high-resolution episcopic microscopy (HREM) volumetric images of E14.5 knockout mouse embryos. In this example, the cross-hairs define the region of a vascular abnormality in a Fam46-/- mouse embryo specimen (DMDD5400). The DMDD consortium have labelled this phenotype as an ‘additional anastomosis between intracranial vertebral arteries’. B) The supporting eHistology image for the section plane shown in A) highlights multiple anatomical components that researchers should additionally consider when evaluating phenotype image data. The web tool we have delivered enables image matching between DMDD and eHistology resources, such that for any given section through a DMDD image volume the closest match from the eHistology (Kaufman) atlas can be retrieved.

In addition to the *eHistology* web-visualisation we have developed JSON *web-services* (see emouseatlas.org/emage/search/json.html) that allow external resources to “discover” matching views through the mouse embryo to correspond to data they may hold. For example, the Wellcome-funded Deciphering the Mechanisms of Developmental Disorders (DMDD) resource (Mohun et al., 2013) delivers phenotype annotated high-resolution image data for mutant mouse embryos. The DMDD interface provides orthogonal views through the embryo data and the *eHistology* web-service API can be used to discover a closely matching annotated view of the high-resolution histology (Fig. 5). We anticipate that this interface will provide a key service for the similar International Mouse Phenotyping Consortium (IMPC) resource and expression atlases.

## 6 Tutorials of development

**Fig. 6:**
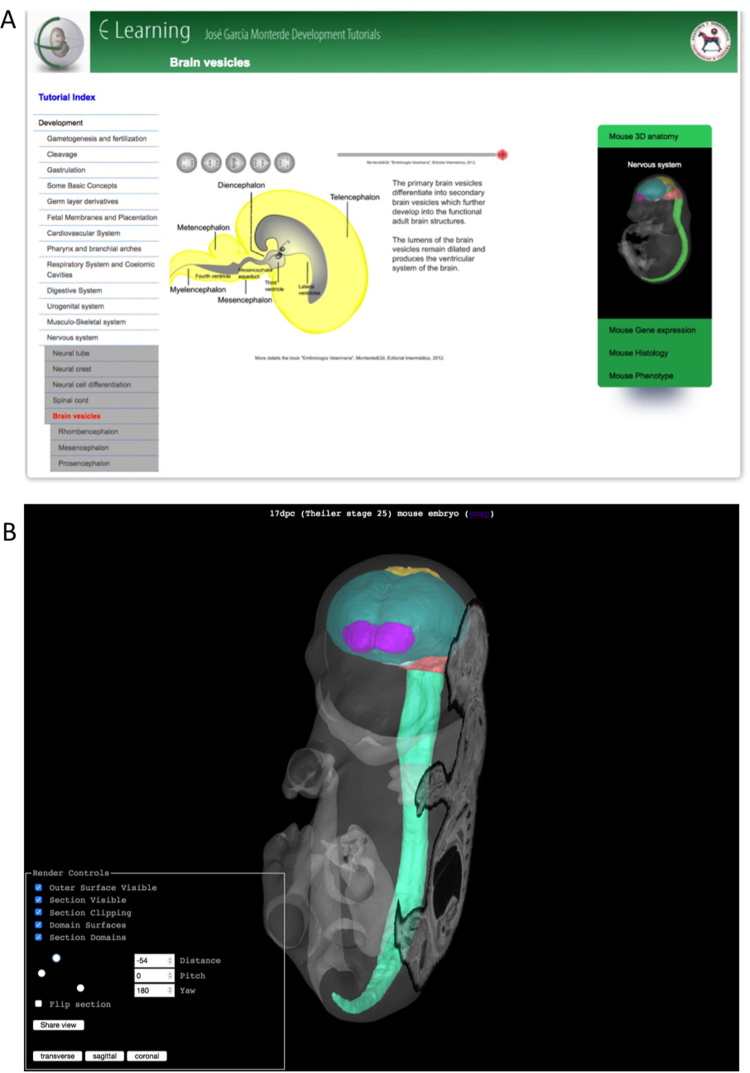
eLearning. eLearning combines animations that enable conceptual understanding of key principles of embryonic development with 3D visualisations of embryo anatomy. A) An animated tutorial is presented in the central panel. The accordion selection tool to the right of the page links to accompanying 3D visualisations. There are additional tabs in the accordion selection tool that link to: EMAGE gene expression patterns associated with developing organ systems; the cellular-resolution eHistology atlas; and DMDD (DMDD.org.uk) phenotype queries. B) The eLearning 3D viewer shows a 3D surface reconstruction of an embryo model, combined with a section through the 3D volume. In this example, the developing nervous system is delineated. By doing so, this viewer enables the detail provided on section to be shown in the context of the 3D anatomy. The navigation tools allow a user to change the section plane. There are additional options to: view/hide the clipping plane through the surface reconstruction; view/hide the section plane through the volumetric image; view/hide the outer surface; view/hide the anatomical domains.

We have recently developed a new *eLearning* resource that provides short and interactive vignettes in embryo (primarily vertebrate) development, from gametogenesis through to organogenesis (Fig. 6). The resource describes the development of various anatomical systems including the cardiovascular system, nervous system, and musculo-skeletal system, and additionally introduces core developmental biology concepts such as gastrulation, placentation, and the formation of the germ layers. The current *eLearning* content are the tutorials produced by Professor José García Monterde of the University of Córdoba, and the presentation from collaboration between Professor Monterde and the eMouseAtlas project.

The resource combines illustrative animations of embryonic development with interactive 3D visualisations of mouse developmental anatomy. The *eLearning* tutorials link through to the EMAGE, EMAP, and *eHistology* resources thus enabling users to find, for example, gene expression patterns associated with developing organ systems. In collaboration with DMDD we additionally provide queries across the DMDD (DMDD.org.uk) database of embryonic lethal phenotypes. These queries utilise a mapping between: 1) EMAPA anatomy ontology terms used by eMouseAtlas; and 2) the Mammalian Phenotype (MP) ontology used by DMDD, and are a means of finding genes that are critically important in the development of specific anatomical components. We envisage the *eLearning* resource as being of great interest for developmental biology students and junior researchers who are unfamiliar with the anatomical nomenclature and who wish to find out more about the underlying principles of mammalian development.

## 7 Limitations of eMouseAtlas resource

The eMouseAtlas programme has pioneered and developed a wide range of tools to visualise and analyse spatial data in an explicit atlas context. However we recognise a number of limitations to the existing resource both in terms of the resource functionality and the resource content. In terms of functionality we need better tools to enable community engagement in terms of data submission and curation. This will be addressed as a core objective of the ELIXIR-UK Biomedical Atlas Centre. In terms of data-content there is a specific gap in annotation of placental anatomy. For this we are developing a cellular-resolution *placental atlas* in collaboration with Professor Myriam Hemberger (Babraham Institute, Cambridge) as part of the Wellcome Trust funded DMDD programme, and this will include annotation on key sections of E9.5 and E14.5 placental specimens From an EMAGE perspective, there is a further need to 3D spatially map sparse section data such as that provided by Eurexpress. Whilst we have had great success in wholemount projection mapping, the Eurexpress and other section-based datasets require an automated method that can map a set of sections into the appropriate space in a 3D model. This method needs to be able to reliably map the ~500 K images of *in situ* data. Related to this issue is a need to integrate the wholemount projection data and the mapped 3D data. There is a complexity in that the spatial similarity scores are substantially different between 2D and 3D datasets. This is because the spatial similarity scores utilises the Jaccard index - a set theory measure of intersection / union – and these are substantially different between 2D and 3D datasets. From a mapping perspective, this is a relatively easy process, however the schema of the EMAGE database needs to be adapted to be able to associate multiple (2D and 3D) mappings with a single entry. Finally the EMAGE resource will need to evolve to enable capture of new techniques for acquiring the spatial-distribution of gene-expression especially single-cell RNA-Seq.

## 8 Discussion

The recent extensions to the Mouse Atlas resource described here are part of a process to develop a community portal to capture and archive key mouse developmental data that would otherwise be lost. *eHistology* and *eLearning* are two good examples of this process with the capture of important knowledge and images. Other examples are the ingestion of data from EurExpress (no longer funded), EmbryoExpress (no longer supported) and the bringing in of the MRI atlas from Caltech (Ruffins and Jacobs, 2011), the MRC NIMR limb atlas (deLaurier at al, 2008), the kidney atlas from EuReGene (now defunct resource) and the pancreas (Hörnblad et al., 2015). The Mouse Atlas resource is now fully part of the ELIXIR-UK node and the future developments will include the extension of the community pages to allow community upload and publication of relevant framework data.

The open source and open access nature of the eMouseAtlas resource lends itself to data integration and extension. Our recent development of a common web portal interface that allows queries across the anatomical atlas, the *eHistology* atlas, and the EMAGE gene expression database greatly increases data accessibility and enables data-sharing with other existing database resources. A perennial issue with such resources is to ensure that the underlying data remain citable and accessible in the long term. A recent study (Attwood et al., 2015) has shown that of 326 biomedical web resources accessible in 1997, 60% were dead and a further 14% archived. The reality is that no web-resource can be considered as a reliable long-term repository for essential research data because of the vagaries of resource funding. The only truly long-term repositories are those associated with long-term institutions and in particular national libraries and Universities. We are therefore using the University of Edinburgh DataShare (datashare.is.ed.ac.uk) to record all of the atlas models and histological data with each dataset acquiring a Digital Object Identifier (DOI) ensuring that these data, and the mechanism to cite them, will be available in perpetuity. The eMouseAtlas resource will continue to evolve as part of the ELIXIR (www.elixir-europe.org) framework and we are a component of the ELIXIR-UK node (www.elixir-uk.org/) with a key objective to enable spatial data interoperability through integration of anatomical atlases that exist for other vertebrate species such as human, mouse, chick, sheep, and pig. A major advantage of this approach is that the cross-species infrastructure will enable comparative analysis between species and molecular data to be incorporated into larger, organism level models. It is anticipated that the introduction of spatial data and anatomical atlases into the ELIXIR infrastructure will enable systems biology approaches to be applied to the mouse, and will have far reaching impact for the management of spatial data from other species.

## Footnotes

1 The distinction between EMAP and EMAGE is historical. The MRC-core funded programme generated 3D reconstructions of key post-implantation stages of development, and these were used as the basis of the Edinburgh Mouse Atlas Project (EMAP) atlas models *and* as a means of archiving gene expression patterns in the Edinburgh Mouse Atlas Gene Expression (EMAGE) database.
